# Home-based management of fever in rural Uganda: community perceptions and provider opinions

**DOI:** 10.1186/1475-2875-6-11

**Published:** 2007-01-26

**Authors:** Xavier Nsabagasani, Jesca-Nsungwa-Sabiiti, Karin Källander, Stefan Peterson, George Pariyo, Göran Tomson

**Affiliations:** 1 Uganda Programme for Human and Holistic Development (UPHOLD), Nakawa House Box 40070, Kampala Uganda; 2 Department of Sociology, Makerere University, Box 7062 Kampala, Uganda; 3 Division of International Health (IHCAR), Karolinska Institutet, 17176 Stockholm, Sweden; 4 Department of Pharmacology and Therapeutics, Makerere University, Kampala, Uganda; 5 Child Health Division, Ministry of Health, Kampala, Uganda; 6 Institute of Public Health, Makerere University, Kampala, Uganda; 7 Medical Management Centre (MMC), Karolinska Institutet, 17176 Stockholm, Sweden

## Abstract

**Background:**

Uganda was the first country to scale up Home Based Management of Fever/Malaria (HBM) in 2002. Under HBM pre-packaged unit doses with a combination Sulphadoxine/Pyrimethamin (SP) and Chloroquine (CQ) called "HOMAPAK" are administered to all febrile children by community selected voluntary drug distributors (DDs). In this study, community perceptions, health worker and drug provider opinions about the community based distribution of HOMAPAK and its effect on the use of other antimalarials were assessed.

**Methods:**

In 2004, four focus group discussions with mothers and 11 key informant interviews with drug sellers, drug distributors and health workers were conducted in Kasese district, western Uganda. This was complemented by three months of field observations.

**Results:**

Caretakers concurred that they were benefiting from the programme. However, according to the information from the DDs and health workers, many caretakers perceived HOMAPAK as a drug of lower quality only meant for first aid. Caretakers also expressed need for other drugs to treat other childhood diseases. The introduction of HOMAPAKs was said not to affect the sale of other allopathic antimalarial drugs in the community. DDs expressed concerns about lack of incentives and facilitation such as torches, gumboots and diagnostic equipment to improve their performance.

**Conclusion:**

HBM is well appreciated by the community. However, more efforts are needed to improve uptake of the strategy through systematic community sensitization and community dialogue. This study highlights the potential of community based volunteers if well trained, facilitated and integrated into a functioning local health system.

## Background

In 2000, the African heads of states committed themselves to the 'Abuja targets' of increasing the number of children with malaria receiving timely and appropriate treatment to 60% [1]. Experimental studies on Home Management of Malaria (HMM) have reduced child mortality and morbidity [2,3]. HMM is now being scaled up in sub-Saharan Africa for prompt access to appropriate dosages within 24 hours of symptom onset [4,5]. Uganda was the first country to adopt HMM and scale it up on a national level as the "Home Based Management of fever strategy" (HBM) [4]. Pre-packaged unit doses of a combination of sulphadoxine/pyrimethamine (SP) and chloroquine (CQ) under the name "HOMAPAK" are distributed to caretakers with febrile children by volunteer community based drug distributors (DDs). At the time of the study first line treatment in Uganda had just changed from CQ alone to a combination of SP and CQ, and HBM was used as a vehicle to disseminate the new first line treatment.

Starting in 2002, HBM was first pilot tested in 10 districts and by the end of 2003 there was a nation-wide scale up. Prior to introduction of HBM, the community effectiveness (CE) of malaria treatment, i.e. the cumulative proportion of fever cases treated promptly with a recommended antimalarial drug using correct dosing and duration, had been shown to be less than 10% [6]. A follow-up evaluation 18 months into the HBM intervention indicated that 25% of febrile children used HOMAPAK [7]. Although this is a considerable improvement it is still far from the Abuja target set for 2005 of 60% of children with fever receiving appropriate treatment within 24 of symptom onset. While pre-HBM studies identified the lack of attention to local fever illness perceptions as a potential barrier to uptake and utilization of HBM [8], post-implementation studies on communities' and health workers' perceptions of the HBM strategy remain unknown. Few studies have studied the formation and implementation of new malaria treatment policies [9,10], and none has so far explored how the policy of community based antimalarial drug distribution diffused into the community.

The aim of this paper was to explore community perceptions, health worker and drug provider opinions of community based distribution of pre-packed antimalarials (HOMAPAK) and its effect on management of fever and use of other antimalarials.

## Methods

### Study area and population

Kasese is located in western Uganda at the foot of Rwenzori Mountains on the border of the Democratic Republic of Congo. The people are mainly subsistence farmers of the Bakonzo tribe (Kasese District, 2000). The district population density is 220/km^2 ^and 89% lives in rural areas. Malaria is hyper-endemic and the under-five mortality rate has been estimated at 170/1000 – mostly caused by malaria and pneumonia [11]. The district has 3 hospitals and 67 health centers scattered in the valleys below the steep hills. 67% of the Kasese population live within 5 kms of a health centre [12]. Kasese was one of the pilot districts for the HBM intervention and implementation started in 2002. The study was conducted two years after HBM implementation in Kanyatsi and Kitholu, the first parishes to receive the HBM intervention.

### Data collection

To explore people's perceptions, experiences, knowledge and opinions about HBM and HOMAPAKs, focus group discussions (FGDs) were viewed as the most appropriate method [13]. Four homogenous FGDs were held with purposively selected groups of young and old mothers ranging between 6–12 per group as they were perceived to have sufficient knowledge and experience on the issues we wanted to address [13]. A skilled social scientist moderator assisted by a note taker, both fluent in the local language, managed the FGDs which lasted on average 45 minutes and were digitally recorded. A thematic guide was used which included management and treatment practices of febrile under-fives, types of medicines used, sources of care sought, perceptions about HOMAPAK efficacy in fever management and opinions about HBM in general.

While the FGDs focused on the perceptions and attitudes about HBM and HOMAPAKs, 11 key informant (KIs) interviews were also performed by two of the authors (XN, JN) to obtain in-depth information on the malaria management situation in the area, the process and progress of implementation, as well as the apparent achievements and challenges faced. The KIs were purposively selected based on their position in the community, their role in implementation, or because of the special information they possessed based on their involvement in childhood fever management. KIs included three health workers from the local health centre in Kanyatsi parish and from Kagando hospital, where most caretakers in the study community are usually referred when their children become severely sick. KI interviews were also performed with four drug sellers and four DDs who were identified with the help of the local leaders.

Health workers provided information on the link between the DDs and the health facilities, the kind of facilitation they provided to the DDs, their experiences about health seeking behavior for fever for the under fives after the introduction of HOMAPAK, their opinions about the strategy and their assessment of DDs performance. Drug sellers from three villages located in the study parishes were purposively selected based on their proximity to drug distributors and having stocks of antimalarial drugs. Drug sellers were asked to share experiences on the effect that HOMAPAK introduction had had on their business and their opinions on the HBM strategy. DDs were asked to share their experiences, achievements and challenges in the distribution of HOMAPAK. When the information became repetitive for each of the categories, interviewee recruitment stopped.

To complement FGD and KI interview findings, one of the authors (KK) undertook daily field observations and informal discussions for a 3 month period in 2004 and spent much time in the homes of some DDs discussing their situation and observing their activities. Rich information was also yielded through prolonged transect walks together with local community members. Peculiar, exciting and everyday mundane activities were recorded in a field diary [14].

### Data analysis

The FGDs were tape recorded, transcribed and translated into English by the FGD moderator. Data from other sources were recorded manually in notebooks and field diaries. Thereafter, the data was systematically coded and analysed manually for content. Recurrent and emerging themes were identified and organized into meaningful categories and sub-categories [15]. Relevant quotations were extracted and some have been presented verbatim. For comprehensiveness, data from the different data collection techniques was triangulated to validate and complement the findings from each of the sources [16]. The findings are presented using a thematic approach whereby responses from different respondents are integrated under the same theme.

### Ethical clearance

The study was approved by the Higher Degrees Research and Ethics of Makerere Medical Faculty, Uganda and the Regional Ethics Committee of the Karolinska Institutet, Sweden (Dnr 02-373). Permission was obtained from the district authorities and local leaders. There was informed consent of the people who volunteered to participate in the study.

## Results

The main findings of the study were (1) communities had not received sufficient information to enable them to understand the rationale of home based management of fever (2) the community's high appreciation of HOMAPAK as the most accessible, free and prompt treatment of hot body which has reduced the prevalence of severe malaria among under fives (3) the perception of HOMAPAK as a "light drug" of lower quality which can not treat severe malaria and not matching with some children's blood (4) the concern of focusing on one disease instead of an integrated approach for managing multiple conditions (5) the lack of community ownership and facilitation of DDs as critical barriers to the sustainability of the programme with motivated DDs.

In terms of preparation, a national communication strategy [17] was developed but never fully implemented. As per the HBMF implementation guideline [4], community mobilization and sensitization was supposed to complement the activities to select and train DDs.

The DDs were given job-aids to explain to mothers how to administer the drugs and when to go for referral. In practice only caregivers visiting the DDs received information through face-to-face counselling. Similarly, the leaflets with drug information enclosed in the HOMAPAK boxes were only accessed by those who had already brought the children to a DD for treatment. In addition a few posters were displayed mainly in the clinics, and village sensitization meetings were only held at the beginning during selection of the DDs.

Respondents reported that HBM made a substantial contribution to the management of malaria in the community. Overall, caretakers in FGDs and KIs concurred that provision of HOMAPAKs by DDs makes the drug easily accessible, hence reducing treatment delays and risks of development of severe malaria with convulsions.

*"It [HOMAPAK] has helped in reducing ekikangararo (convulsions). In the past, children would die a lot but now the rate has reduced. We honor HOMAPAK" (FGD, old mothers)*.

Most caretakers during FGDs confidently argued that HOMAPAK is effective if administered in time:

*"In fact in this village we have mothers who do not like taking their children early enough for treatmentthey even blame other mothers who attend quickly to child's illness. It is always out of negligence and carelessness. And for your information it is such people who will preach that HOMAPAK does not work well on children" (FGD, old mothers)*.

However, a few caretakers in FGDs remarked that HOMAPAKs are not effective, especially when the malaria is severe.

*"HOMAPAKs can only be good when the child is not very sick. You need strong drugs if malaria is high" (FGD, old mothers)*.

Both DDs and health workers agreed that some people in the community perceived HOMAPAK as a weak drug and only useful for non severe malaria. Some of the caretakers who perceive HOMAPAK as weak drug may avoid going to the drug distributor when their children have fever.

*"You know people in our community think that drugs provided in the community are very weak. They refer to them as 'omubatsi owahesi' meaning 'drugs of lower quality'. They believe that the drugs provided in the clinics and drug shops are better" (a DD)*.

Health workers and DDs also confirmed that community members sometimes bypassed the DDs and sought treatment directly from health facilities or drug shops where they perceived the drugs to be 'stronger'. This was also confirmed by the drug sellers who reported that the introduction of HOMAPAKs had not affected their sales and many people still prefer drugs from their shops:

*"For me I have not seen how it has affected my business. People still come to me because I have all the drugs they need like panadol, aspirin and some antibiotics. There are some people who come after failing with HOMAPAK and I give them quinine. People prefer my drugs because I have sugar coated chloroquine which most people prefer to the bitter one" (a drug seller)*.

In addition, DDs and caretakers reported that there were some children whose fever does not respond to HOMAPAK treatment, which has led to mistrust of the drug. While health workers were aware of the problem with drug resistance, caretakers on the other hand attributed this to the mismatch between the child's blood and HOMAPAK.

*"The resistance to HOMAPAK nowadays is a common problem. When there is no effect for those who have used the drug, they lose confidence and spread the rumours to others" (a health worker)*.

*"We did not go for HOMAPAK again because we suspected that the blood of the child does not work with HOMAPAK or there was another complication or probably the drug was not effective" (a caretaker in clinic)*.

The community members were not satisfied with the fact that HBM only brings one type of treatment and demanded more drugs for the management of other childhood illnesses.

*"I would not suggest to change it but it [HOMAPAK] should be accompanied with another pack containing cough tablets, painkillers and those of flu" (FGD, young mothers)*.

DDs also confirmed that caretakers expected them to have a variety of drugs to treat other childhood diseases in addition to malaria. In addition, both caretakers and DDs expressed the need for antimalarial drugs for the adults because if not treated, their malaria was believed to spill over to the children.

*"HOMAPAKs are unpopular because they do not provide them for the old people. Only children are given the drugs and yet sometimes the fever attacks the whole family at once. Instead of going to the DDs, they will go to the drug shops where they will get drugs for everyone" (a DD)*.

Both caretakers and DDs were concerned that HOMAPAK was not being provided with Oral Rehydration Salt (ORS) which, according to them, is essential for the management of most of childhood illnesses. DDs acknowledged the fact that caretakers had extensive knowledge about ORS and considered it to be important also for fever management. Reasons mentioned included that it restores water in the body, gives the child strength, adds blood, reduces the body temperature, stops the diarrhea, and supports the other drugs the child is taking. They further argued that ORS would be especially necessary in combination with HOMAPAK which is perceived to dehydrate children.

*"I use ORS for rehydration purposes. Also we want ORS because Fansidar requires a lot of fluids. Also if the child is too weak, he/she needs ORS and drinks. So we need ORS to be part of the HOMAPAK. That is why mothers prefer going to Kagando [Hospital] because in Kagando, they are given ORS especially to a child who is vomiting and has diarrhea" (FGD, old mothers)*.

*"If the DDs were given ORS they would be more respected in the community" (KI, community Leader)*.

Lack of facilitation of DDs was raised by different respondents. Respondents maintained that DDs lacked tools to make their work easier and both DDs and caretakers agreed that diagnostic equipment would improve diagnosis and hence attract more caretakers of febrile children. Equipment such as thermometers and microscopes were cited as critical.

*"We need thermometers to check the level of temperature. During the training we were told that we should always establish the temperature and when we find that it is too high we refer the children to the health facility" (a DD)*.

Apart from diagnostic equipment, DDs said they needed facilitation with lighting to enable them identify the colored packs at night, gumboots to guard against snake bites when visiting homes, soap to wash hands before handling medicines and containers to keep medicine safely. The concern about lack of DD facilitation was frequently raised also by mothers.

*"What happens is when you call him [DD] he comes and checks your child and after another parent may call him in another place. So if it is at night, these people [DDs] suffer in the darkness and you know our place is hilly so they need lamps and paraffin" (FGD, old mothers)*.

Moreover, DDs and caretakers were concerned about the lack of remuneration and recognition by local government authorities for the work performed. According to the DDs' coordinator, the work load at the health centers hampers health staff from interacting with and supervising the DDs in the community. This problem was also confirmed during informal discussions with DDs and community leaders who mentioned the lack of payment and supervision as a great barrier to motivation.

*"What makes somebody happy is the stomach. Although the DDs may not be complaining directly, lack of allowances is demotivating them. They will do their work at their own pace. They cannot leave their gardens to attend the ailing children" (Community Leader)*.

## Discussion

This study highlights four key findings in relation to the HBM strategy. First, in line with Kilian's predictions from pre-HBM studies [18] HOMAPAK is highly appreciated in the community as the most accessible, affordable and prompt treatment of hot body. Second, although HOMAPAK is popular, it is perceived as good only for non-severe malaria and not "matching" with some children's blood, especially if the treatment fails. Third, there is concern about focusing on one disease instead of an integrated approach for managing multiple conditions. Fourth, lack of community ownership, facilitation and incentives for DDs are critical barriers to a sustainable programme with motivated DDs.

### Perceptions of drug efficacy

The discussion about the mismatch between the child's blood and the drug is a reflection of the emerging perceptions about the HOMAPAK innovation. The biomedical view is that if the child does not improve after administration of HOMAPAK, the possible reasons for the treatment failure could be caused by inappropriate dosing, drug resistance, or that the cause of the fever may be another disease than malaria. The caretakers view this failure differently; they suspect either a mismatch between the drug and the child's blood, or that HOMAPAK is a weak drug. This implies that they may not give HOMAPAK again to a child who did not respond to this treatment the first time which may partly explain the 25% utilization-rate of HOMAPAK for fever observed in a quantitative evaluation study [7].

To understand community perception of efficacy these issues have to be viewed in light of the cultural system which provides a more satisfactory explanation of efficacy than the biomedical view [19]. People's evaluation of efficacy is based on experience and many expect highly effective drugs to make symptoms disappear immediately [20]. Having used a drug that worked is a strong reason as to why the users selected the drug again [21]. A study in Burkina Faso also showed that severity of disease and perceived effectiveness of the treatment were the most important determinants of low utilization of the community health workers [22].

This implies that HOMPAKs will need to be updated to contain drugs with high biological efficacy [23], to minimize treatment failures. Furthermore, there is need to understand and communicate on key community perceptions on the intervention as they emerge. For instance if the child who has been given HOMAPAK does not improve this may not be an "incompatibility with the child's blood" but the illness could be caused by another illness than malaria and that there is need for referral for reassessment [24].

### One disease versus integrated management approach

Whereas HBM stresses the importance of treating all fevers as malaria, caretakers are aware that fever is a symptom of several illnesses and often treat symptoms as separate diseases with a variety of drugs [25,26]. Consequently, they request a wider scope of treatments to be available from the DDs. Furthermore, the large symptom overlap between pneumonia and malaria also raises concern for possible mistreatment and aggravation of a child with pneumonia if treated with antimalarials alone [27]. Hence there is need to modify HBM to a more integrated approach, addressing communities' own priorities while still realizing that not every condition can be treated in the community [28]. Providing ORS through the DDs seems safe and is in line with the WHO/UNICEF recommendation to integrate community management of diarrhoea and pneumonia in HBM strategies [29]. Integrating antibiotics for pneumonia and ORS for diarrhea in the community based management of childhood fevers would fit both with community expectations and epidemiological needs. The policymakers will benefit from additional evidence from the African setting on the feasibility, cost and effectiveness of integrating community management of pneumonia and diarrhea into existing single disease management programmes such as Home Management of Malaria [30].

### Policy and community approach

HBM was designed to follow a systematic consultative and participatory process involving different stakeholders at the national, district and community levels [4]. However, there has been limited time for consulting the community, discussing their needs, understanding their perceptions and agreeing on the role they were supposed to play in implementing and sustaining of the programme. The rapid scale up did not allow for community dialogue and community empowerment [31,32]. Unless these limitations are addressed, the result may be continued low utilization of HOMAPAKS, especially among the poorest that are often most difficult to reach [7]. Furthermore, the sustainability of DDs will remain a challenge as long as there is little community commitment to support DDs.

While a desirable, participatory process in every village is not compatible with rapid national scale-up – a balance needs to be reached. This study implies that if HBM is to succeed and be sustained, more investment should be made in an iterative process of community consultation and establishment of interventions that are well integrated into government structures. This iterative process would include localized studies to not only help understand the local context, perceptions and construction of local medical knowledge [33], but also detect concerns emerging from the implementation experiences [34]. In this context, qualitative methods have the potential to identify effects of context, cultural values and local perceptions on the service delivery and utilization [35,36]. Information from such studies will be useful in the design of communication packages to optimize diffusion of national drug policy and enhance community adoption of new interventions such as HBM. Community sensitization is also another vital input and should include informing the communities about the possibilities of resistance-induced treatment failure, side effects and the need of referral in case of no improvement. As evident from this study much more powerful, locally appropriate behaviour change communication needs to be implemented to increase uptake and address misconceptions on a new intervention like HBM [8].

### Supporting DDs for sustainable good performance

The drug distributors did not feel well supervised, facilitated or recognized by the local government. The importance of recognition, training, supervision and incentives for community volunteers have been highlighted in several studies [37-39]. Although the HBM guidelines recommended giving incentives to the DDs [4,5] this has not been realized. Due to high level of unemployment, people volunteer with hope that they will be remunerated eventually [40]. But long-term pure voluntary work is hard to sustain and in countries with already severely constrained health budgets, monetary incentives for an additional cadre of health workers may not be a realistic option [41]. Still, the use of volunteers without any form of incentives could be a liability not only to the sustainability of the intervention, but also to DD performance, as motivational factors are a crucial component in the performance equation [42]. This effectively means that a strong community health programme requires a strong health system, and innovative incentive programmes to keep up the quality and motivation among community volunteers. If well trained, facilitated and integrated into the local health system, DDs could contribute substantially to prompt and appropriate management of acute febrile illness.

### Methodological considerations

Like with most qualitative studies the magnitude of the problems identified could not be established, such as use of the HOMAPAKS in relation to other drugs and the extent of referral. Another limitation was that the subject was sensitive in case of some respondents such as DDs and health workers who were reluctant to discuss weaknesses in the approach since they would share part of the blame. By triangulating several qualitative methods with different respondents, it was possible to identify and confirm pertinent issues. Observations complemented the other findings and provided contextual information which was useful in the analysis. Although there were no serious contradictions between the different methods of data collection, some categories of respondents were more elaborate on some information than others. For instance for DDs and health workers, the perception that HOMAPAK is a light drug was a big concern, whereas the caretakers themselves did not dwell on it so much.

## Conclusion

This study has demonstrated that the community appreciates the contribution of home based management of fever (HBM). This is an important prerequisite in the strengthening of HBM. This achievement has been realized as a result of the availability of drugs at the community level through the services of DDs. If well trained, facilitated and integrated into the local health system, DDs could contribute substantially to prompt and appropriate management of acute febrile illness. There is a need to operationalize methods to address the perceptions of drug efficacy and community expectations in systematic and strong information/behaviour change communication to increase uptake and use of drugs supplied by DDs, as well as aspects of retention and performance of DDs. There is also a need for an iterative approach whereby current efforts are evaluated and improved over time which requires a close link between policy and research [43]. In this context, qualitative methods have the potential to identify effects of context, cultural values and local perceptions on the service delivery and utilization [35].

## Authors' contributions

1. XN conceived the study, participated in the planning and implementation of the study, analyzed the data, drafted and finally revised the manuscript

2. JNS conceived the study, participated in the planning and implementation of the study, analyzed the data, commented on the draft manuscript and participated in the revision of the manuscript

3. KK conceived the study, participated in the planning and implementation of the study, analyzed the data, commented on the draft manuscript and participated in the revision of the manuscript

4. SP conceived the study, participated in the planning of the study, analyzed the data, commented on the draft manuscript and participated in the revision of the manuscript

5. GP conceived the study, participated in the planning of the study, analyzed the data, commented on the draft manuscript and participated in the revision of the manuscript

6. GT conceived the study, participated in the planning of the study, analyzed the data, commented on the draft manuscript and participated in the revision of the manuscript

All authors read and approved the final manuscript.
